# Development and Validation of an LC–MS/MS Method for Quantifying Phytohormones Related to Tomato Shelf Life

**DOI:** 10.3390/foods14234040

**Published:** 2025-11-25

**Authors:** Muhammad K. Hakeem, Haneen Abufarajallah, Maryam Abushahab, Gamilah Abdulgabar, Hind Alneyadi, Shaikha Alnaqbi, Sampathkumar Elangovan, Iltaf Shah

**Affiliations:** Department of Chemistry, College of Science, United Arab Emirates University (UAEU), Al Ain P.O. Box 15551, United Arab Emirates

**Keywords:** LC-MS/MS, phytohormones, liquid chromatography-mass spectrometry, tomato, plant hormone, method validation

## Abstract

Phytohormones are key signaling molecules that regulate plant growth, stress adaptation, and fruit ripening. However, their low abundance and structural diversity complicate accurate quantification in food matrices. This study presents a validated LC–MS/MS method for the simultaneous detection of seven phytohormones in tomato fruit, including two synthetic analogs that mimic natural auxins and cytokinins. Method optimization focused on extraction efficiency, solid-phase cleanup, and mobile phase composition, achieving high recovery (85–95%) and reduced matrix effects. Chromatographic separation was performed on a C18 column, with detection by triple quadrupole mass spectrometry in MRM mode. The method demonstrated excellent linearity (R^2^ > 0.98), precision, and robustness, with detection limits as low as 0.05 ng/mL for abscisic acid and 6-benzylaminopurine. Validation followed US-FDA and EC 2021/808 guidelines, ensuring regulatory compliance and analytical reliability. Analysis of tomato samples from five geographic origins revealed significant differences in phytohormone profiles, particularly in abscisic and salicylic acids, highlighting the method’s ability to capture biologically and agriculturally relevant variation. This workflow offers a sensitive, transferable platform for monitoring bioactive compounds in tomatoes and other food crops, supporting post-harvest quality assessment and food metabolomics research.

## 1. Background

Phytohormones, critical regulators of plant growth and development, have been extensively studied in recent years due to their vital roles in mediating responses to environmental stimuli and controlling key physiological processes [1,2]. Phytohormones, also known as plant hormones, are signaling molecules that regulate various physiological processes in plants. These small molecules act as messengers, facilitating communication within the plant and in response to external factors. The intricate balance and precise regulation of phytohormone levels are fundamental for the overall well-being of plants [3,4]. Among the diverse classes of phytohormones, cytokinins, auxins, gibberellins, abscisic acid, ethylene, and brassinosteroids, along with strigolactones, jasmonates (including jasmonic and salicylic acids), nitric oxide, and systemin peptides, play vital roles in plant growth, development, and stress adaptation [3,5,6]. Tomato (Solanum lycopersicum) is one of the most important globally due to its high consumption rate and economic significance [7,8]. Recent advances in phytohormone research in tomatoes have highlighted their roles in fruit development, stress responses, and yield optimization [9,10,11,12,13]. Understanding the dynamics of phytohormones in tomatoes is crucial for optimizing crop yield, quality, and resilience. Additionally, since the tomato plant is considered a model organism, insights obtained from tomato studies can be applied to other plants in the Solanaceae family, which comprises approximately 3000–4000 species across nearly 90 genera. This highly diverse family includes perennial trees and herbaceous animals that thrive in a wide range of terrestrial habitats, from deserts to rainforests. Members of Solanaceae hold great importance in human civilization, serving as food crops (e.g., potato, tomato, pepper, eggplant, pepino, naranjilla, and tamarillo), ornamentals (e.g., Petunia, Datura, Schizanthus, and some Solanum species), and medicinal plants (e.g., Tobacco, Atropa, Hyoscyamus, and Mandragora) [14,15,16]. Thus, a comprehensive analysis of phytohormones in plants provides valuable insights into agricultural practices and crop improvement.

Plants, lacking a nervous system, utilize alternative mechanisms for signaling to regulate organ development and growth [17,18]. Environmental signals such as light, temperature, and moisture directly affect the synthesis, catabolism, and translocation of plant hormones [19]. Quantifying multiple plant hormones simultaneously is necessary to gain a comprehensive understanding of plant physiology [20,21]. Phytohormones are categorized into various groups, including auxins, cytokinins (CK), gibberellins (GA), salicylates (SA), Abscisic acid (ABA), brassinosteroids (BR), ethylene (ETH), jasmonates (JA), and strigolactones (SL) [22,23,24,25]. These classes are complemented by additional signaling molecules such as nitric oxide (NO), systemin peptides, and karrikins, which function synergistically with classical phytohormones to regulate growth and stress responses [6,26]. Auxins are derivatives of indole, CKs are derivatives of adenine, ABA is a sesquiterpene, BRs are polyhydroxy steroids, ETH is a simple alkene, GAs are tetracyclic diterpenoid acids, SLs are terpenoid lactones derivatives of carotenoids, and JA is a fatty acid derived from linolenic acid [27,28,29,30,31]. Similarly, salicylic acid and jasmonates serve as central regulators of plant defense and signaling cascades [32]. Some other known identified plant growth regulators include plant-derived peptide hormones such as Systemin and CLE peptides [33,34], karrikins [35,36], nitric oxide (NO) [37], and polyamines [38,39].

This study focuses on analyzing five essential phytohormones: Indole-3-acetic acid, Isopentenyl adenine, Gibberellic acid, Salicylic acid, and Abscisic acid, along with two synthetic molecules 2-Naphthalene acetic acid and 6-benzylaminopurine. While plants do not naturally synthesize 2-Naphthalene acetic acid and 6-benzylaminopurine, these compounds mimic the functions of natural auxins and cytokinins, respectively, and are widely used in agricultural research to better understand plant growth and development. 2-NAA promotes cell elongation and root initiation, whereas 6-BAP has been shown to enhance plant resistance to abiotic stresses and improve seedling growth under adverse environmental conditions [40,41,42]. The method developed in this study aims to address concerns regarding the presence of phytohormones, including synthetic molecules, in tomato samples and to assess whether these compounds are inadvertently introduced during cultivation.

Several methods are available for quantifying phytohormones in plants, including biological approaches such as bioassays and immunoassays [43], as well as chemical approaches like gas chromatography/mass spectrometry (GC-MS) and N-doped carbon nanotube-reinforced hollow fiber solid-phase microextraction (N-doped CNTs-HF-SPME) [44,45,46]. Other methods include a UV detector which is a chromatography detector used to measure the visible or ultraviolet light absorbed by a sample, or a Photodiode array (PDA) detector, which utilizes reverse optics and makes it more versatile since it uses all wavelengths of the spectrum [47,48,49]. Other methods include Fluorescence detectors (FLD) that work by passing a beam of light through the sample which excites electrons causing the emission of light from the sample [50,51]. All these methods are considered cheap, accessible, and accurate options. However, they have higher limits of detection and quantification compared to LC-MS.

Analytical techniques for the quantification of phytohormones have evolved over the years. The coupling of liquid chromatography with tandem mass spectrometry (LC-MS/MS) has emerged as a superior analytical technique for quantifying phytohormones [46,47,48,52,53]. The integration of LC with mass spectrometry provides an extremely sensitive and quantitative tool for detecting and identifying phytohormones, overcoming the limitations of other techniques like GC-MS. The introduction of MS-based analytical techniques has significantly improved the sensitivity, accuracy, and practicality of plant hormone measurements. LC-MS/MS, in particular, allows for high separation performance and the detection of trace-level plant hormones in complex matrices without the need for derivatization. This study aims to fill existing gaps in the understanding of phytohormonal dynamics in tomatoes by developing and optimizing a method for the simultaneous quantification of multiple phytohormones using LC-MS/MS. This method not only enables the detection of both natural and synthetic hormones with high sensitivity and specificity but also addresses the challenge of quantifying phytohormones in complex plant matrices. Although previous research has focused on the quantification of individual phytohormones or small sets of hormones [8,20,52,54,55], these studies often fail to capture the complex interactions and synergies between multiple phytohormones during fruit development. This study presents an optimized method for the simultaneous analysis of seven phytohormones, including two synthetic molecules (2-Naphthalene acetic acid and 6-Benzyl aminopurine), using LC-MS/MS. To our knowledge, no previous studies have investigated the comprehensive analysis of phytohormones in tomatoes using this method. The development, validation, and application of this LC-MS/MS method aim to provide a more complete understanding of the role of phytohormones in the shelf life and stability of tomatoes, with potential applications in improving agricultural practices and crop management.

## 2. Materials and Methods

### 2.1. Chemicals & Reagents

LC-MS grade methanol, formic acid, and acetic acid were obtained from Supelco (Darmstadt, Germany) and Fluka (Buchs, Switzerland). Milli-Q-Water was obtained from In-house (UAE University, Al Ain, United Arab Emirates). Indole-3-acetic acid (100%), Isopentenyl adenine (98.5%), 2-Naphthalene acetic acid (95%), 6-Benzyl aminopurine (99%), Gibberellic acid (90%), Salicylic acid (99%), Abscisic acid (98%), Salicylic acid (99%) and Salicylic acid d4 (internal standard) were purchased from Sigma-Aldrich (St. Louis, MO, USA).

### 2.2. Preparation of Standard Solution

The preparation of standard solutions is a critical step in ensuring precision and accuracy in phytohormone analysis. Phytohormone standard solutions were prepared by dissolving approximately 5 mg of each compound in 5 mL of LC-MS/MS grade methanol to produce a stock solution of 1 mg/mL. Methanol was chosen as the solvent to ensure the solubility and stability of the phytohormones, providing a reliable foundation for LC-MS/MS analysis. Calibration standards were prepared by diluting the stock solution with a 50:50 methanol-water diluent for linearity analysis. This process followed established methodologies for LC-MS/MS analysis, which have been widely applied in phytohormone quantification studies [56,57]. This standardized approach ensures consistent and reliable quantification of phytohormones. Methanol’s use as the solvent further enhances the stability of phytohormones, forming the basis for robust analytical performance.

### 2.3. Tomato Plant Cultivation, Collection, and Sample Preparation

To investigate the phytohormonal profiles in tomatoes, a diverse range of tomato samples was sourced from local markets across different geographic regions, including the United Arab Emirates (UAE), Malaysia, Iran, India, and Holland. This broad geographic distribution of market-sourced tomatoes allowed for a comparative analysis of phytohormonal profiles across various growing environments and agricultural practices. By employing market-based sampling, our study ensures a comprehensive analysis of phytohormonal dynamics across a wide array of tomato varieties and growing conditions. The sample preparation process began with the careful removal of the tomato fruit skin, followed by the extraction of flesh, excluding seeds. To enhance the efficiency of the subsequent steps, the obtained material was finely ground using a mortar and pestle, with liquid nitrogen introduced during grinding to preserve the sample’s integrity and facilitate the creation of a solid form. The prepared homogenized material was weighed immediately after grinding under liquid nitrogen to approximately 1 g fresh weight (FW) for extraction. The frozen powder was transferred directly into extraction tubes while maintaining cryogenic conditions to prevent analyte degradation. Because the samples were obtained as fresh market fruits, the results were normalized to a fresh-weight basis (ng/g).

For the extraction of phytohormones, 0.05 mL of Salicylic acid D4 (500 ng/mL) was added as an internal standard, and the mixture was vortexed to ensure thorough mixing. Salicylic acid D4 was selected due to its structural similarity to the target analytes. Although not all phytohormones share the exact structural features of Salicylic acid, the D4-labeled form was effective in compensating for sample matrix effects, thus aiding in the reliable quantification of the targeted phytohormones.

A comparative analysis was conducted to evaluate the effectiveness of the sample preparation method. This approach involved an initial extraction with acetonitrile containing 1% acetic acid, followed by dilution with a mobile phase of methanol and water (35:65). Acetonitrile was used in the extraction to facilitate protein precipitation and enhance the extraction of phytohormones from the samples. This optimized method resulted in extraction efficiencies ranging from 85% to 95%, consistent with high recoveries reported for acidified organic extraction workflows and higher than several earlier single-step or less-optimized protocols in comparable plant matrices, where recoveries for some analytes can be appreciably lower (e.g., ~60–70% depending on matrix and cleanup) [21,55,58]. Additionally, this approach reduces matrix effects and supports improved LC–MS/MS sensitivity, in line with prior SPE-based phytohormone methods [52,54]. The deliberate exclusion of acetonitrile from the mobile phase minimized ion suppression, enhancing the ionization efficiency of target phytohormones, particularly gibberellic acid and abscisic acid. Moreover, the simplicity of this method reduces both sample handling complexity and analytical runtime, facilitating efficient high-throughput analysis. To further enhance the reliability of the extraction process, Solid-Phase Extraction (SPE) was incorporated using a C18 cartridge. This clean-up step effectively removed non-polar interferences, concentrating the target analytes to ensure maximum sensitivity and specificity during LC-MS/MS analysis. The resulting solution was subjected to centrifugation at 3000× *g* for 10 min at 4 °C to separate the components. After centrifugation, 1 mL of the supernatant was carefully extracted and mixed with 1 mL of the mobile phase. This step was crucial for optimizing chromatographic separation and minimizing matrix interferences. This extraction method, though simple, has been validated for accurate phytohormonal quantification in tomatoes and provides a robust alternative to more complex extraction techniques [59,60].

### 2.4. LC-MS/MS Analysis and Method Development

Liquid chromatography-tandem mass spectrometry (LC-MS/MS) analysis was performed using a SHIMADZU Nexera X2 system comprising an LC-30AD binary pump and an LC-MS 8060 Shimadzu mass spectrometer (Shimadzu Corporation, Kyoto, Japan). Chromatographic separation was achieved using a ZORBAX Eclipse Plus C18 column (4.6 × 100 mm, 3.5 µm, Agilent Technologies, Santa Clara, CA, USA). The mass spectrometer operated in Electrospray Ionization (ESI) mode, utilizing both positive and negative ionization. The mobile phases consisted of formic acid (0.01%, *v*/*v*) in water and methanol (35:65, *v*/*v*). The mobile phase flow rate was set to 0.5 mL/min, and the injection volume was 10 µL. The autosampler temperature was maintained at 5 °C, while the column temperature was carefully controlled at 30 °C. Prior to validation, method optimization was conducted to enhance chromatographic resolution and sensitivity. The mobile phase composition was optimized by varying the methanol-water ratio (20:80 to 80:20, *v*/*v*), with a 35:65 ratio yielding the best separation and minimal ion suppression. Column temperature (tested at 25 °C, 30 °C, and 35 °C) was set at 30 °C to ensure optimal resolution, while flow rate (0.5 mL/min) provided the best balance of sensitivity and resolution. Collision energies (CE) for each analyte were optimized individually to maximize ionization efficiency, resulting in a 20–30% improvement in signal intensity. These optimizations ensured robust and reliable performance across all analytes, making the method suitable for high-throughput analysis of phytohormones in tomato matrices.

### 2.5. MS Tuning Procedure and Parameters

The MS parameters were optimized by injecting diluted standards in full scan followed by selecting the best multiple reaction monitoring (MRM), which means the best precursor and product ions for the determination of phytohormones. The triple quadrupole instrument utilized argon as the collision gas in the collision cell, supplied at high purity and regulated internally by the system to ensure reproducible fragmentation efficiency. The main stock solution of plant hormones was used for the preparation of tuning solution by combining the phytohormone solutions with a 50:50 mixture of methanol and water, resulting in a 100 ng/mL concentration. This solution is then subjected to injection into an LCMS/MS system. The LCMS/MS analysis is carried out using MRM to optimize the Q1, Q3, and fragmentation parameters. MRM enables precise monitoring of specific mass transitions, enhancing the accuracy and sensitivity of the analysis. The optimized final CE values and tuning parameters are listed in Table 1. The results were used as one quantifier to quantify the analyte, and the remaining results were qualifiers to confirm the presence of the compound.

### 2.6. Method Validation

The validation of the LC-MS/MS method was carried out following established guidelines from the US Food and Drug Administration (US-FDA) to ensure the robustness and reliability of the method [61]. Several critical validation parameters were assessed, including linearity, limit of detection (LOD), limit of quantification (LOQ), accuracy, precision, and recovery. Quality control (QC) samples were prepared at three concentrations: low (10 ng/mL), medium (50 ng/mL), and high (200 ng/mL) to evaluate intra- and inter-day accuracy and precision. Acceptance of the inter-day precision accuracy happened when the experimental concentrations were obtained around 15% of the actual concentration, while acceptance of the lower limit of quantification (LLOQ) occurred after obtaining results within a 20% limit range. The quality control data that was obtained after analysis was used to calculate the inter/intra-day precision (% CV) data using the following equation:

$$\left[\%\;\mathrm{CV}=\;(\frac{standard\;deviation}{mean})\times 100\right]$$

The inter/intra-day accuracy (% recovery) was calculated using following equation:

$$\left[\%\;\mathrm{Accuracy}\;=\;(\frac{mean\;value}{nominal\;value})\times 100\right]$$

Whereas Nominal value is calculated concentration. It is determined by:

$$Nominal\;value=\frac{Analyte\;area}{Internal\;standrad\;area}$$

The importance of the validation process lies in its capacity to demonstrate that the analytical method is relevant for the intended purpose and to ensure that the obtained values are close to the analyte content present in actual samples [62,63]. As part of the method validation, we also evaluated the ion ratio for each phytohormone by comparing the intensity of the quantifier (primary) to qualifier (secondary) product ions monitored in multiple reaction monitoring (MRM) mode. This parameter is used as an additional identity-confirmation criterion: for each phytohormone, the ion ratio observed in sample extracts must agree within ±30% of the ratio obtained for the corresponding calibration standard, as recommended by the US-FDA and EC 2021/808 validation guidelines [64]. This evaluation ensures the robustness of the method and its compliance with the required standards for analyte confirmation and selectivity. To evaluate robustness, several key parameters were varied to assess their impact on method performance. The column temperature was adjusted from 30 °C to 35 °C, and recovery remained within 80–120%, indicating the method’s stability under temperature variations. The flow rate varied by ±0.05 mL/min from the optimized flow rate (0.5 mL/min), with recovery and precision remaining within acceptable limits. Additionally, the mobile phase composition was slightly altered (±5% methanol-water ratio), and recovery remained within the desired range, confirming method accuracy. The injection volume varied by ±10 µL from the optimal 10 µL, with stable recovery values. Lastly, sample preparation conditions were modified by adjusting the extraction volume (±1 mL), and recovery remained consistent. These variations showed that the method is robust and reliable for phytohormone quantification, even with minor experimental adjustments.

## 3. Results and Discussion

### 3.1. Method Validation Results

The method employed for the analysis of phytohormones involved measuring their retention time and relative abundance (intensity). The results in Table 2 display the method validation parameters, including the linearity range, R^2^ values, LOD, LOQ, intra-day and inter-day accuracy, and precision data for each analyte. The outstanding regression value (R^2^ = 0.99) suggests a high degree of correlation between the concentration of phytohormones and their corresponding signal intensities. This is a crucial aspect of method validation, ensuring accurate and reliable quantification across a range of concentrations. Spiking was performed using 10 µL of each phytohormone standard at a concentration of 100 ng/mL, ensuring consistency across all samples. The quality control concentrations (Low quality control (LQC), Medium quality control (MQC), and High quality control (HQC)) help evaluate how matrix components influence the analyte signal. By comparing the observed concentration in spiked samples to the nominal concentration, the method accounts for potential matrix interferences that could suppress or enhance the analyte signals. Accuracy was assessed by comparing the spiked concentrations with the observed concentrations, while precision was evaluated using the relative standard deviation. Both accuracy and precision were assessed through interday and intraday analyses, with results found to be satisfactory (accuracy within 80–120% and precision <20%). The findings indicate that the method is highly sensitive, accurate, and precise, providing valuable insights.

**Figure 1:**
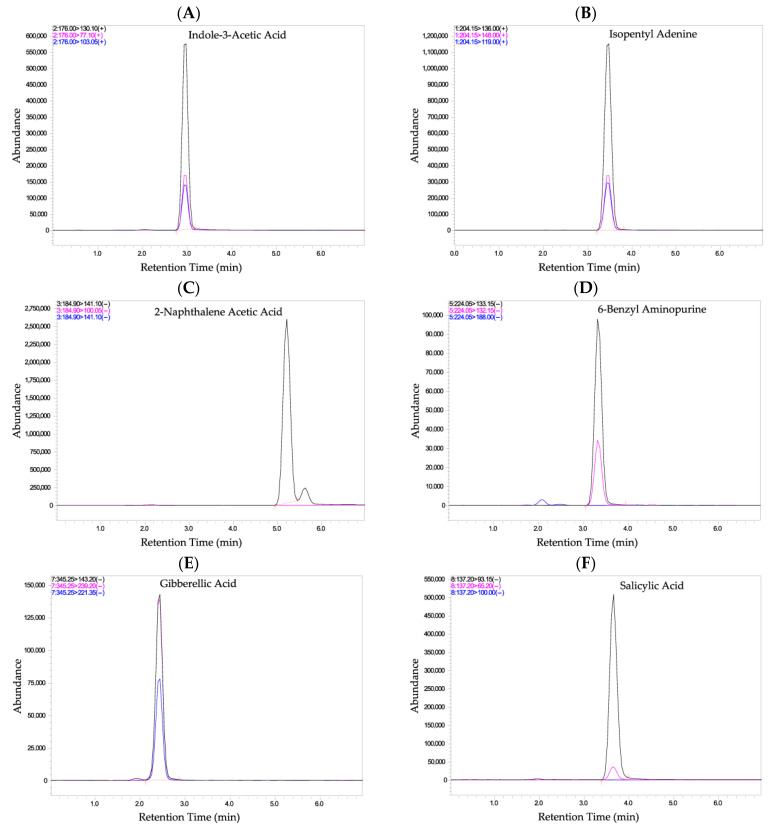

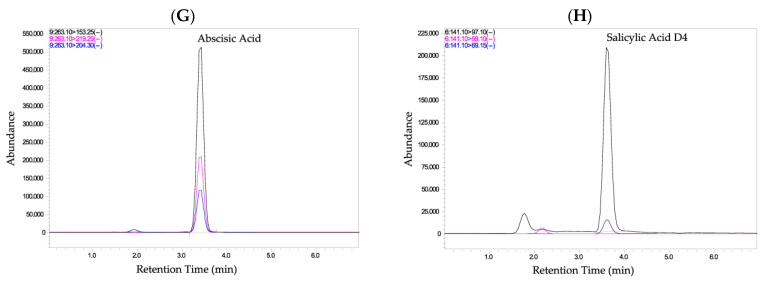
Individual Chromatograms showing the detection of phytohormones and internal standard. (**A**) Indole-3-acetic acid; (**B**) Isopentenyl adenine; (**C**) 2-Naphthaleneacetic acid; (**D**) 6-Benzylaminopurine; (**E**) Gibberellic acid; (**F**) Salicylic acid; (**G**) Abscisic acid; and (**H**) Salicylic acid-D4 (internal standard). Compound identification and quantification were confirmed by their specific MRM transitions, demonstrating overall method selectivity and accuracy.

Figure 1 displays chromatogram that visually represent the phytohormones along with the internal standard. Each hormone is depicted as a well-defined peak at its specific retention time. The clear separation of peaks is essential for accurate identification and quantification of individual compounds. Matrix peaks, which could arise from other substances in the sample, are appropriately excluded from the analysis. Each compound was accurately identified and quantified through its specific MRM transition, ensuring reliable distinction from potential interferences. Selectivity in this method therefore refers primarily to the ability of the LC–MS/MS system to distinguish and quantify the target analytes in the presence of matrix components through mass-spectrometric selectivity, rather than complete chromatographic resolution.

To further confirm the selectivity, the method was validated by analyzing blank samples. The absence of significant signals in blank samples assures that the peaks observed in the chromatograms are indeed associated with the phytohormones and not interference from the matrix. Additionally, the response in blank samples was compared to that of spiked LLOQ samples which were prepared from linearity solution, and the difference was within the satisfactory range (less than 30%). This further validates the method’s ability to accurately quantify phytohormones in the presence of potential sample matrix effects. The combination of HPLC and MS/MS components provides a powerful tool for detecting and confirming the presence of hormones in matrices (such as tomato fruit). This technique offers high specificity and sensitivity, and it is widely applied for the simultaneous identification of phytohormones and other metabolites.

**Figure 2:**
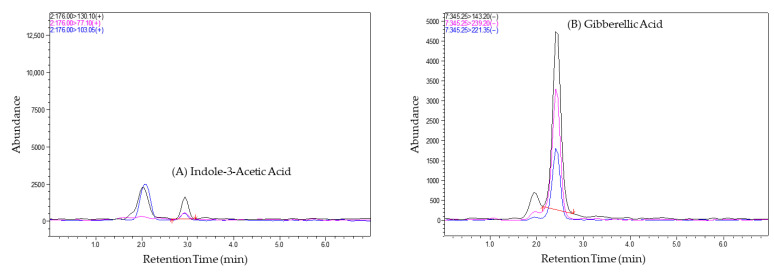

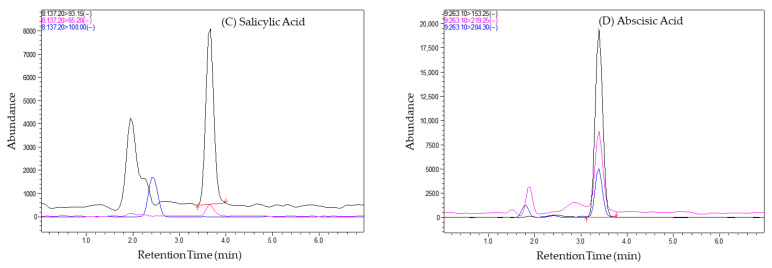
Chromatograms of tomato samples showing the separation of phytohormones (**A**) Indole 3-acetic acid, (**B**) Gibberellic acid, (**C**) Salicylic acid, (**D**) Abscisic acid, with distinct retention times, enabling their identification and quantification. Black represents the precursor ion (Q1), and blue and pink represent the product ions (Q3).

The term “chromatographic conditions” refers to the specific set of parameters and techniques employed during the chromatographic analysis. The chromatographic conditions facilitated the separation, identification, and quantitation of phytohormones in tomato fruit samples. Using chromatographic separation, this method could effectively separate the phytohormones during the analysis of tomato samples, as a difference was observed between them in terms of their retention times, as shown in Figure 2 below. Different phytohormones have distinct chemical properties, leading to variations in their retention times. The chromatograms (Figure 2) visually represent these differences, allowing for the easy identification of individual phytohormones based on their elution times.

### 3.2. Sample Analysis

**Figure 3:**
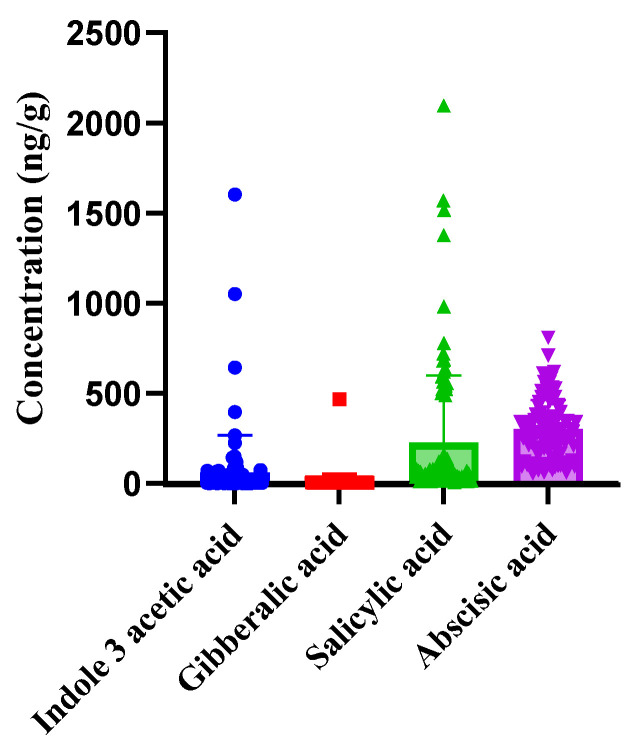
The plot shows the overall concentrations of detected phytohormones in tomato samples (= 5 countries), with error bars indicating standard deviations, highlighting the significant variation in phytohormone levels across samples.

**Figure 4:**
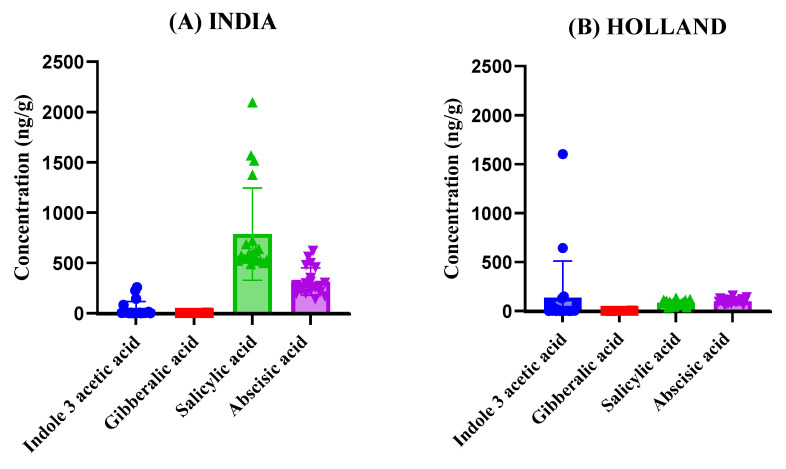

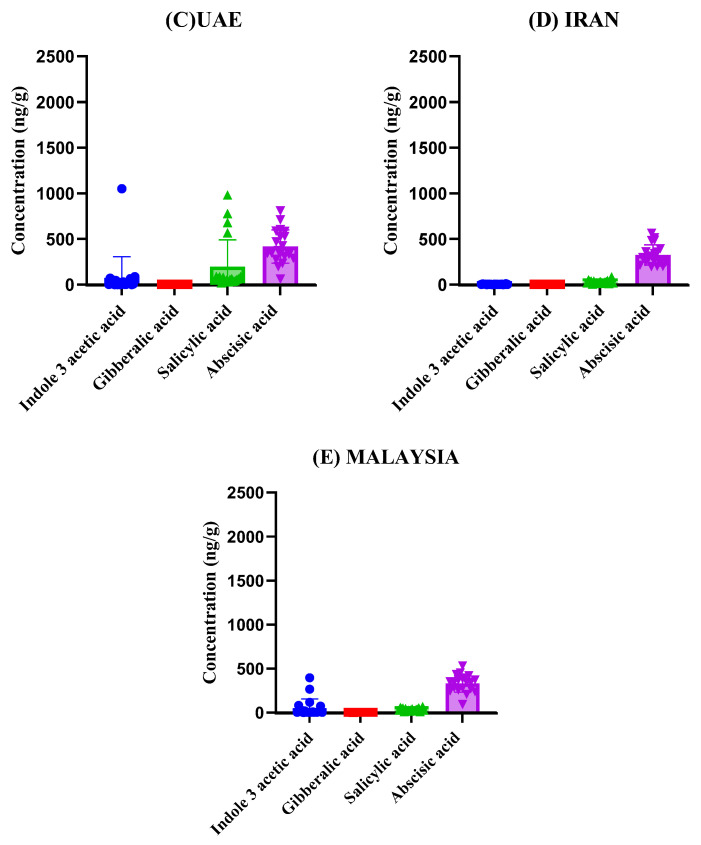
Comparison of phytohormone concentrations across tomato samples from different countries (**A**) India, (**B**) Holland, (**C**) United Arab Emirates, (**D**) Iran, (**E**) Malaysia. Subfigures (**A**–**E**) show the variation in Indole-3-acetic acid, Gibberellic acid, Salicylic acid, and Abscisic acid levels among the samples.

The validated LC-MS/MS method was employed to analyze the phytohormonal content in tomato samples from each of the five countries. The results, as shown in Figure 3, revealed the presence of four phytohormones in all samples: Indole-3-acetic acid, Gibberellic acid, Salicylic acid, and Abscisic acid. Each of these hormones play a specific role in plant growth, development, and response to environmental stimuli. The concentrations of these phytohormones varied among the samples, with Abscisic acid consistently found in the highest concentrations across all samples, suggesting its prominent role during fruit maturation and ripening. On the other hand, Indole-3-acetic acid had the lowest concentration among the detected phytohormones indicating its limited presence during the final stages of tomato ripening. The results are expressed in nanograms per gram (ng/g) of fresh weight, as this unit accurately represents the concentration range observed in tomato matrices and ensures readability, consistent with previous LC–MS/MS studies on phytohormone quantification in fruits [65,66]. The plot in Figure 3 exhibits notable variation in concentrations of detected phytohormones among tomato samples. This variability could be due to several factors such as genetic differences among the tomato plants, environmental conditions, or other external factors. All data points were visualized using bar plots with overlaid individual values and error bars (Figure 3 and Figure 4), allowing the identification of any potential extreme values. All data points were reviewed for consistency, and no exclusions were made unless justified by confirmed technical error. Statistical analysis, including one-way ANOVA, confirmed that the differences in phytohormone concentrations among samples were statistically significant (*p* < 0.05), further underscoring the influence of external factors on phytohormonal levels. The existence of significant variation aligns with findings that have previously been reported in the literature [52].

The graphs presented in Figure 4 provide a detailed comparison of phytohormone concentrations across tomato samples from each of the five countries. By presenting the results side by side, we can observe variation in phytohormonal levels among samples from different geographical origins, which may reflect general differences in local growing environments, tomato cultivars, or post-harvest handling. As the tomatoes were market-sourced, detailed information regarding specific agricultural practices, genetic subspecies, nutrient management, or pesticide use was not available; therefore, these factors are discussed in a general, interpretative sense based on existing literature rather than from direct measurement. The results reveal substantial variation in the concentrations of Indole-3-acetic acid, Gibberellic acid, Salicylic acid, and Abscisic acid among the different countries. For instance, tomatoes from United Arab Emirates exhibited the highest levels of Abscisic acid on average followed by India, suggesting that tomatoes cultivated in these regions may undergo more significant stress responses or ripening processes compared to those from other regions. In contrast, tomatoes from Holland displayed relatively lower concentrations of Abscisic acid, potentially indicating differences in storage conditions or the timing of harvest. Salicylic acid concentrations showed notable fluctuations across samples, with tomatoes from India displaying the highest average concentrations. This could reflect differences in post-harvest handling or the specific cultivars used. Gibberellic acid levels were comparatively lower in tomatoes from Malaysia, indicating a less pronounced role in their ripening process compared to the samples from Iran, where higher levels were recorded (Figure 4). Although the developed LC–MS/MS method was optimized and validated for the simultaneous analysis of seven phytohormones (including two synthetic analogs, 2-Naphthalene acetic acid and 6-Benzylaminopurine), only four compounds were detected above the quantification limit in the analyzed tomato samples. The remaining three analytes were either absent or present below the method’s limit of detection (LOD). This outcome likely reflects the natural hormonal composition of mature tomato fruit rather than a limitation of the analytical method. The developed method remains fully capable of detecting all seven hormones in matrices or developmental stages where they occur within quantifiable ranges.

Statistical analysis confirmed significant differences in phytohormone concentrations among the countries (*p* < 0.01), suggesting that regional origin may be associated with measurable variation in hormonal composition. However, as the tomato samples were commercially obtained, specific details regarding genotype, cultivation practices, planting schedules, or climatic parameters were unavailable. Thus, the observed differences are interpreted as region-associated trends rather than causally linked outcomes, with genetic and agronomic factors also likely contributing to the observed variability. These findings not only highlight the robustness of the LC-MS/MS method in detecting and quantifying phytohormonal levels but also contribute to the growing body of literature on how external factors influence tomato ripening and shelf life.

## 4. Conclusions

In this study, we successfully developed, optimized, and validated an LC-MS/MS method for the precise quantification of key phytohormones in tomatoes. By optimizing the phase composition, we achieved improved separation and resolution, and the method adhered to stringent US Food and Drug Administration (FDA) guidelines, ensuring precision, accuracy, specificity, and linearity. The MS tuning process optimized compound detection and established ideal conditions for quantification. This validated method demonstrated high sensitivity with reduced sample run times and simplified extraction procedures, contributing to its potential for more efficient analysis in various laboratory settings. Chromatograms confirmed the presence of phytohormones, showing distinct peaks at specific retention times. The analysis of tomato samples from various geographic origins revealed significant variations in phytohormone levels, with Abscisic acid generally present at higher concentrations and Indole-3-acetic acid at the lowest. An exception was observed in tomatoes from India, which displayed comparatively elevated Salicylic acid levels and slightly lower Abscisic acid concentrations, potentially reflecting heat- and stress-related physiological responses commonly associated with tropical growing conditions. These findings indicate that such variability may be influenced by general factors such as cultivar characteristics, growing climate, and post-harvest physiology. Previous studies have shown that Abscisic acid and ethylene act as key maturity and senescence-regulating hormones, with ABA known to promote ripening by enhancing ethylene biosynthesis and activating ripening-related gene expression [67,68,69]. Although these processes were not directly monitored here, the observed variation among countries aligns with such known post-harvest physiological mechanisms. This validated LC–MS/MS method not only enabled accurate quantification of endogenous phytohormones and revealed distinct compositional differences among samples from different regions but also confirmed the absence of the two synthetic phytohormone analogs, 2-Naphthalene acetic acid and 6-Benzylaminopurine, in all analyzed tomato samples. This outcome supports the method’s sensitivity and reinforces its applicability for monitoring potential exogenous hormone residues during cultivation or post-harvest handling. This study provides a methodological and analytical insight into the roles of key phytohormones in fruit ripening and post-harvest quality. The developed method offers a valuable tool for further research into phytohormonal interactions and their effects on agricultural products. Additionally, this approach can be adapted to quantify bioactive compounds in other plant species, potentially advancing the study of plant growth regulators in agricultural science.

## 5. Future Prospects

This study has successfully developed and validated an LC-MS/MS method for quantifying key phytohormones in tomatoes. However, there are several avenues for future research to further enhance the understanding and application of this method. The developed technique can be adapted to quantify phytohormones in a wider range of agricultural crops. Expanding its use to other fruits and vegetables will enable comparisons across different species and provide deeper insights into plant growth regulation in diverse agricultural settings. Additionally, while the current sample preparation method is effective, there is potential for further optimization to minimize sample volume and improve throughput, which would enhance the scalability of the method for large-scale analyses.

Further research could involve investigating phytohormone dynamics over the entire growth cycle of tomatoes, from seedling to post-harvest, to better understand how these hormones influence various stages of development and ripening. Moreover, integrating this method with other analytical techniques, such as transcriptomics or proteomics, could provide a more comprehensive understanding of the molecular mechanisms that regulate phytohormonal activity in tomatoes and other plants. Additionally, future studies could explore the environmental and agricultural factors that influence phytohormone concentrations, such as soil composition, irrigation practices, and the impact of climate change on plant growth and fruit quality. Studying the effect of post-harvest handling and storage conditions on phytohormonal content could also help optimize post-harvest management strategies and improve shelf life and product quality.

## Figures and Tables

**Table 1:** The operating parameters and conditions of Mass Spectrometry.

Compound Names	Q1 (*m*/*z*)	Q3 (*m*/*z*)	Retention Time (min)	Dwell Time (ms)	Q1 Pre-Bias (V)	CE (V)	Q3 Pre-Bias (V)	ESI Polarity
Indole-3-acetic acid	176	130.1	2.921	100	−17	−15	−22	**+**
	176	77.1		100	−16	−42	−15	
	176	103.1		100	−16	−29	−23	
Isopentenyl adenine	204.2	136	3.545	100	−20	−15	−20	**+**
	204.2	148		100	−10	−13	−14	
	204.2	119		100	−10	−31	−11	
2-Naphthalene acetic acid	184.9	117.1	5.07	100	22	11	26	**−**
	184.9	100.1		100	20	34	38	
	184.9	141.1		100	22	10	20	
Indole-3-Butyric Acid	202.1	134.2	3.523	100	22	16	20	**−**
	202.1	133.2		100	22	22	20	
	202.1	132.2		100	22	30	28	
6-Benzyl aminopurine	224.1	133.2	3.381	100	24	23	26	**−**
	224.1	132.2		100	24	32	26	
	224.1	188		100	26	13	22	
Gibberellic acid	345.3	143.2	2.412	100	17	29	13	**−**
	345.3	239.2		100	17	16	30	
	345.3	221.4		100	17	25	22	
Salicylic acid	137.2	93.15	3.096	100	13	21	11	**−**
	137.2	65.2		100	14	28	15	
	137.2	100		100	15	27	26	
Abscisic acid	263.1	153.3	3.34	100	28	12	20	**−**
	263.1	219.3		100	28	14	24	
	263.1	204.3		100	28	20	26	
Salicylic acid D4 (Internal standard)	141.1	97.1	2.901	100	14	21	12	**−**
	141.1	59.1		100	14	12	14	
	141.1	69.15		100	14	30	8	

(Q1: mass parent ion, Q3: mass product ion, Dwell time: Time spent acquiring specific MRM transition, Q1 pre-bias: Voltage needed to promote ionization of precursor ion, Q3 pre-bias: Voltage needed to promote ionization of product ion).

**Table 2:** Method validation parameters including Linearity range, Correlation coefficient (R^2^), Limit of Detection (LOD), Limit of Quantification (LOQ), Intra-day and Inter-day Accuracy and Precision.

Compound	Quality Control	Concentration (ng/mL *)	Linearity Range (ng/mL *)	R^2^	LOD (ng/mL *)	LOQ (ng/mL *)	Intraday		Interday		**Ion Ratio (>±30)**
							Accuracy%	RSD%	Accuracy%	RSD%	
6-Benzyl aminopurine	LQC	0.13	0.129–24.441	0.99	0.05	0.15	106.15	15.55	116.41	17.61	3.34
	MQC	0.261					102.11	10.57	103.45	10.05	
	HQC	1.304					98.5	3.73	104.48	6.39	
Abscisic acid	LQC	0.499	0.411–1.447	0.996	0.05	0.16	90.08	3.84	98.63	8.54	−1.13
	MQC	0.999					115.27	6.5	96.77	7.79	
	HQC	4.994					95.2	3.28	94.25	1.96	
Salicylic acid	LQC	0.513	0.504–107.082	0.995	0.3	0.89	88.73	11.87	98.97	12.95	1.46
	MQC	0.98					94.1	9.51	102.57	4.12	
	HQC	4.913					99.74	0.76	93.83	1.33	
Gibberellic acid	LQC	1.008	1.004–229.010	0.99	0.68	2.05	110.47	20.02	109.9	7.95	−10.53
	MQC	2.015					108.1	6.57	108.62	13.88	
	HQC	10.076					96.43	9.48	100.22	6.55	
Indole-3-acetic acid	LQC	0.997	1.010–214.041	0.997	0.3	0.91	102.56	8.23	86.84	14.3	0.97
	MQC	1.994					107.43	7.96	103.02	6.69	
	HQC	9.972					93.56	2.32	95.58	3.05	
2-Naphthalene acetic acid	LQC	131.681	125.404–24,624.728	0.996	15.27	46.27	93.72	4.15	97.31	5.36	7.03
	MQC	263.361					101.7	2.16	104.05	3.16	
	HQC	1316.806					103.35	2.06	104.98	1.56	
Isopentyl Adenine	LQC	0.062	0.063–13.700	1	50.01	0.04	96.5	7.4	89.25	6.06	−1.88
	MQC	0.124					94.76	3.94	99.2	4.4	
	HQC	0.628					93.76	1.85	95.92	2.07	

RSD is calculated from 6 replicates: LOD=3×SD, LOQ=10×SD; * The experiment is performed by using the Matrix Match method.

## Data Availability

The original contributions presented in the study are included in the article, further inquiries can be directed to the corresponding author.
